# Semantic aware-based instruction embedding for binary code similarity detection

**DOI:** 10.1371/journal.pone.0305299

**Published:** 2024-06-11

**Authors:** Yuhao Jia, Zhicheng Yu, Zhen Hong

**Affiliations:** College of Information Engineering, Zhejiang University of Technology, Hangzhou, Zhejiang, China; Najran University College of Computer Science and Information Systems, SAUDI ARABIA

## Abstract

Binary code similarity detection plays a crucial role in various applications within binary security, including vulnerability detection, malicious software analysis, etc. However, existing methods suffer from limited differentiation in binary embedding representations across different compilation environments, lacking dynamic high-level semantics. Moreover, current approaches often neglect multi-level semantic feature extraction, thereby failing to acquire precise semantic information about the binary code. To address these limitations, this paper introduces a novel detection solution called BinBcla. This method employs an enhanced pre-training model to generate instruction embeddings with dynamic semantics for binary functions. Subsequently, multi-feature fusion technique is utilized to extract local semantic information and long-distance global features from the code, respectively, employing self-attention to comprehend the structure information of the code. Finally, an improved cosine similarity method is employed to learn relationships among all elements of the distance vectors, thereby enhancing the model’s robustness to new sample functions. Experiments are conducted across different architectures, compilers, and optimization levels. The results indicate that BinBcla achieves higher accuracy, precision and F1 score compared to existing methods.

## 1. Introduction

Owing to the non-open source nature of the source code of commercial software, malicious code, and legacy programs, conducting binary code similarity analysis on these software applications holds significant importance in various security applications. These applications include vulnerability search, malware detection, code clone detection, etc. The objective of binary code similarity analysis is to identify structurally and semantically similar code fragments within a target binary code repository based on known binary code snippets. However, binary code is typically represented as assembly code functions obtained through disassembling executable files. Unlike functions in high-level languages, understanding functions in binary code is often challenging. In real-world scenarios, variations in architectures, compilers, and optimization levels can result in substantial changes to binary code. Since binary code lacks a vocabulary of natural language semantics, as found in source code, extracting semantic information presents a challenging task. Before the advent of machine learning in this field, traditional methods relied heavily on control flow graph (CFG) of binary code. However, these methods, while demonstrating a certain level of effectiveness, fail to capture essential features of binary code and are characterized by complex feature extraction processes, high computational overhead, and poor scalability. Moreover, current approaches represent functions as CFG without conducting multi-level feature extraction, thereby failing to acquire precise semantic information about the binary code and resulting in lower accuracy. Binary code embedding represents an emerging approach in similarity analysis, employing neural network models to transform binary code into vector representations. This method not only captures the semantic information of binary code, but also facilitates quantitative analysis of the similarity between corresponding binary codes by computing distances between vectors. Effective code representation helps mitigate the impact of significant assembly code differences resulting from diverse compilation environments. Recent advancements in binary code similarity detection have incorporated pre-trained natural language processing (NLP) models. SAFE [1] utilized Word2Vec to represent binary functions as sequences of assembly instructions, capturing both syntax and semantic information of instructions through BiRNN. Asm2vec [2] generated embeddings for functions based on the paragraph-vector distributed-memory (PV-DM) model. PalmTree [3] introduced the first BERT-based instruction embedding model. UniASM [4] employed unsupervised learning and a pre-trained BERT-based model to generate instruction embeddings. Despite the diverse algorithms employed by these deep learning-based methods, they all adhere to the concept of code embedding.

To further enhance code representation and improve the performance of detection, we employ an enhanced BERT-based pre-training model. This model’s pre-training tasks include masked language model (MLM) and similar function prediction (SFP), specifically tailored for constructing a universal model for binary code representation. Instruction embedding module models assembly functions by treating assembly instructions as tokens, converting each instruction into an embedding vector, and considering binary functions as sentences composed of sequences of instruction vectors. Subsequently, the embedding vectors of instructions are inputted into local semantic module to extract distinct local semantic features. Bidirectional LSTM is utilized to extract features capturing long-distance dependencies, capturing sequential interaction relationships within the vector sequences. When aggregating into functions, self-attention is applied to learn dependencies between instructions, allowing the final function embedding to consider the hidden states of all instructions. Finally, improved cosine similarity and cross-entropy loss are employed to learn distance vectors, understanding relationships among all elements of the distance vectors. This fundamentally enhances the model’s robustness to new sample functions. We conduct binary code similarity detection experiments across different architectures, different compilers, and different optimization levels. The experimental results demonstrate that our novel detection solution, called BinBcla, outperforms previous approaches in terms of accuracy, precision and F1 score.

In summary, the proposed method makes the following contributions:

1. We develop an instruction embedding module, based on an enhanced BERT architecture, to provide dynamic semantic embeddings for binary code instructions. It dynamically adjusts token embedding based on varying contextual information, addressing the challenge of handling polysemy that Word2Vec struggles with.
2. We propose a multi-feature fusion technique that performs feature extraction at different levels on binary code, greatly enriching the semantic feature information of binary code.
3. We propose an improved cosine similarity method that addresses the issue of traditional cosine similarity being unable to distinguish differences between data objects with proportionally changing values in various dimensions, thereby enhancing the performance of model.
4. We conducted experiments on three tasks, and the results indicate that BinBcla achieves better performance compared to existing methods.

The remainder of this paper is organized as follows: Related work section discusses related work in this field. Methodology section introduces our methodology, including instruction embedding and multi-feature fusion. Experimental setup section introduces implementation details. Evaluation section introduces the results of experiments. In conclusion section, we conclude our method and direction for future research.

## 2. Related work

### 2.1 Traditional approaches

Before the application of machine learning, traditional techniques mainly involved dynamic analysis and static analysis. Dynamic analysis methods rely on the premise that binary code sharing similar behavior at runtime exhibits logical similarity. These methods assess binary code similarity by analyzing manually crafted dynamic features. Genius [5], based on the graph isomorphism of CG/CFG, employs graph matching algorithms to validate the equality of two basic blocks. Pewny [6] represents each vertex of CFG using expression trees, calculating the similarity between vertices based on the edit distance between corresponding expression trees. ESH [7] employs theorem-proving to calculate whether two basic blocks of function are equivalent. BinSim [8] performs dynamic slicing through system calls and then employs symbolic execution to assess the equivalence of binary code. BinGo [9] and Blex [10] initialize function context through random sampling values, collecting I/O values to determine the similarity of functions. The drawback of these methods is high computational cost and execution time, rendering them unsuitable for large-scale detection. Static analysis methods, based on differences in the structure of binary code, typically involve converting binary code into graphs and subsequently comparing the similarity of these graphs. Binslayer [11] employs the Hungarian algorithm to enhance graph matching, thereby improving the results for binary functions. BinSign [12] and Kam1n0 [13] use instructions or categorized operands as static features to compute the similarity of binary. BinSequence [14] compares the similarity of two functions by calculating the edit distance between their instruction. XMATCH [15] leverages the graphs and tree edit distance of expression trees for basic blocks to compute the similarity of binary functions. DiscovRE [16] optimizes CFG-based matching by pre-filtering and eliminating unnecessary matching pairs. TEDEM [17] introduces tree edit distance to detect basic block-level code similarity. These methods rely solely on the structure or syntactic features of binary code, neglecting semantic information between instructions, resulting in relatively low detection accuracy.

### 2.2 Learning-based approaches

In recent years, some research studies have applied deep learning. A common approach in these studies involves representing binary functions as numerical vectors through instruction embedding models, and then approximating the similarity between different binary functions using vector similarity [18]. These methods utilize Siamese neural networks to ensure that vectors of logically similar binary functions are closer in distance. Xu [19] proposed Gemini, which relies on graph embedding techniques to generate binary function embeddings through attributed CFG (ACFG) and Structure2vec. INNEREYE [20] and RLZ2019 [21] treat instructions as words and basic blocks as sentences, using Word2Vec to generate function embeddings and LSTM to learn block embeddings. Li [22] introduced a simple BiRNN-based Ins2vec embedding method. Ben [23] proposed a detection method based on Transformer and convolutional neural network (CNN). *α*Diff [24] utilizes TextCNN [25] to directly learn embeddings for binary functions from the raw bytes of the functions. DEEPBINDIFF [26] and Codee [27] employ neural networks to learn vector embeddings for instruction sequences. In the field of unsupervised learning, a notable solution for binary code similarity detection is Asm2Vec [2], which generates instruction sequences from CFG and employs unsupervised learning algorithms to generate embeddings for binary functions. Additionally, Massarelli [28] and Yu [29] investigated methods to learn embeddings for basic blocks of binary functions and utilized GNN to learn embeddings for ACFG of binary functions. Methods based on deep learning have exhibited high accuracy and computational efficiency. Leveraging these advantages, such approaches are well-suited for large-scale detection.

## 3. Methodology

### 3.1 Overview

**Fig 1:**
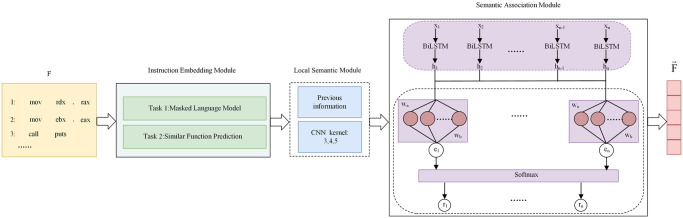
Structure of the overview.

To address the abovementioned challenges, we utilize an enhanced BERT-based instruction embedding model, in which assembly instructions are first transformed into embedding vectors. Subsequently, these vectors are inputted into a Siamese neural network with multi-feature fusion as subnetworks. A self-attention layer is incorporated into the Siamese neural network to obtain weight information corresponding to the instruction vectors. Based on the weights derived from the self-attention, the instruction embeddings are aggregated into a function embedding. Ultimately, a similarity score between the two functions is calculated using improved cosine similarity. The overall structure is illustrated in Fig 1.

### 3.2 Instruction embedding module

The feature extractor of BERT employs Transformer, preserving richer semantic information in word vector representations. This has demonstrated excellent performance across various NLP tasks. Instruction embedding module utilizes the encoder of the Transformer structure to build a neural network model consisting of a multilayer bidirectional encoder. Designed as a universal model for constructing binary code representations, instruction embedding module is capable of vector embedding for each instruction within a function; its pre-training tasks include MLM and SFP.

#### 3.2.1 Masked language model

**Fig 2:**
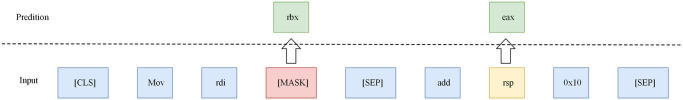
Masked language model.

Assume that *t*_*i*_ represents a token, and the function instruction *I* = *t*_1_, *t*_2_, *t*_3_, ⋯, *t*_*n*_ is composed of a sequence of tokens, as illustrated in Fig 2. For instruction *I*, the following process is applied. First, 15% of the tokens are randomly selected for replacement. Among the masked tokens, 80% are replaced with [MASK], 10% are replaced with another randomly chosen token from the vocabulary, and the remaining 10% are left unchanged. Subsequently, the Transformer is employed to learn predictions and output the probability of predicting a specific token *t*_*i*_ = [MASK] through Softmax:

$$p(\hat{t_{i}}I)=\frac{\text{exp}\left(w_{i}\Theta {\left(I\right)}_{i}\right)}{\sum_{k=1}^{K}\;\text{exp}\left(w_{k}\Theta {\left(I\right)}_{i}\right)}$$ (1)

where $\hat{t_{i}}$ represents the prediction for *t*_*i*_, Θ(*I*)_*i*_ denotes the *i*-th layer vector of the final layer of Transformer Θ when the function instruction *I* is used as the input, *w*_*i*_ is the weight for *i*, and *K* is the number of possible labels for the *t*_*i*_.

#### 3.2.2 Similar function prediction

**Fig 3:**
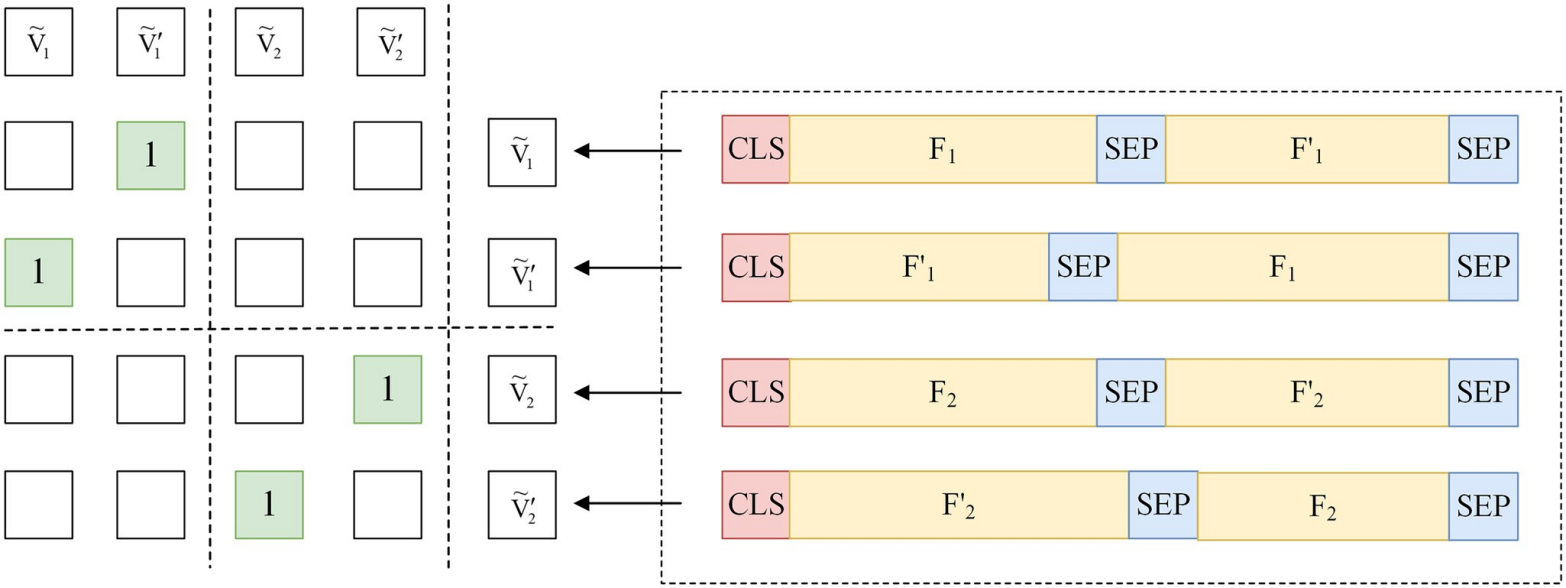
Similar function prediction.

SFP processes each batch of function pairs during data processing, rather than a single function pair. As depicted in Fig 3, each sample of each batch represents a pair of similar functions, such as [CLS] F [SEP] F′ [SEP], where F and F′ denote similar functions. Swapping the two similar functions constructs [CLS] F′ [SEP] F [SEP], which is then appended to the original samples. Consequently, each batch contains an even number of function pairs.

The embedding for the *k*-th function in each batch is denoted as **v**_*k*_ = [*v*_1_, *v*_2_, ⋯, *v*_*d*_], where *d* represents the size of the hidden layer. Subsequently, L2 normalization is applied to each element in the embedding:

$${\tilde{v}}_{i}=\frac{v_{i}}{\sqrt{\sum_{j=1}^{d}{v_{j}}^{2}}}$$ (2)

The normalized function embedding vector, denoted as ${\tilde{v}}_{k}=\left[{\tilde{v}}_{1},{\tilde{v}}_{2},\cdots ,{\tilde{v}}_{d}\right]$, is obtained. Batch normalization is then utilized to construct the embedding matrix $\tilde{V}={\left[{\tilde{v}}_{1},{\tilde{v}}_{2},\cdots ,{\tilde{v}}_{b}\right]}^{T}$, where *b* is the size of batch.

To calculate the similarity between two functions, we take the dot product between the embedding matrix $\tilde{V}$ and its transpose ${\tilde{V}}^{T}$:

$$S=\tilde{V}\cdot {\tilde{V}}^{T}=\left\{s_{ij}\right\},i,j\in \left[1,2,\cdots ,b\right]$$ (3)

The matrix **S**, referred to as the similarity matrix, is defined such that each value represents the similarity between two functions. Smaller angles correspond to more similar vectors. To mitigate the impact of diagonal elements in the similarity matrix, all diagonal elements are set to negative infinity in this paper:

$$S=\tilde{V}\cdot {\tilde{V}}^{T}-\Lambda \left[+\infty \right]$$ (4)

where Λ[+∞] represents the diagonal matrix. Each row of the similarity matrix is processed through a Softmax layer:

$$p\left(s_{ij}\right)=\frac{\text{exp}\left(s_{ij}\right)}{\sum_{k=1}^{b}\;\text{exp}\left(s_{ik}\right)}$$ (5)

where *s*_*ij*_ represents the similarity between the *i*-th and *j*-th functions.

### 3.3 Multi-feature fusion

#### 3.3.1 Local semantic module

**Fig 4:**
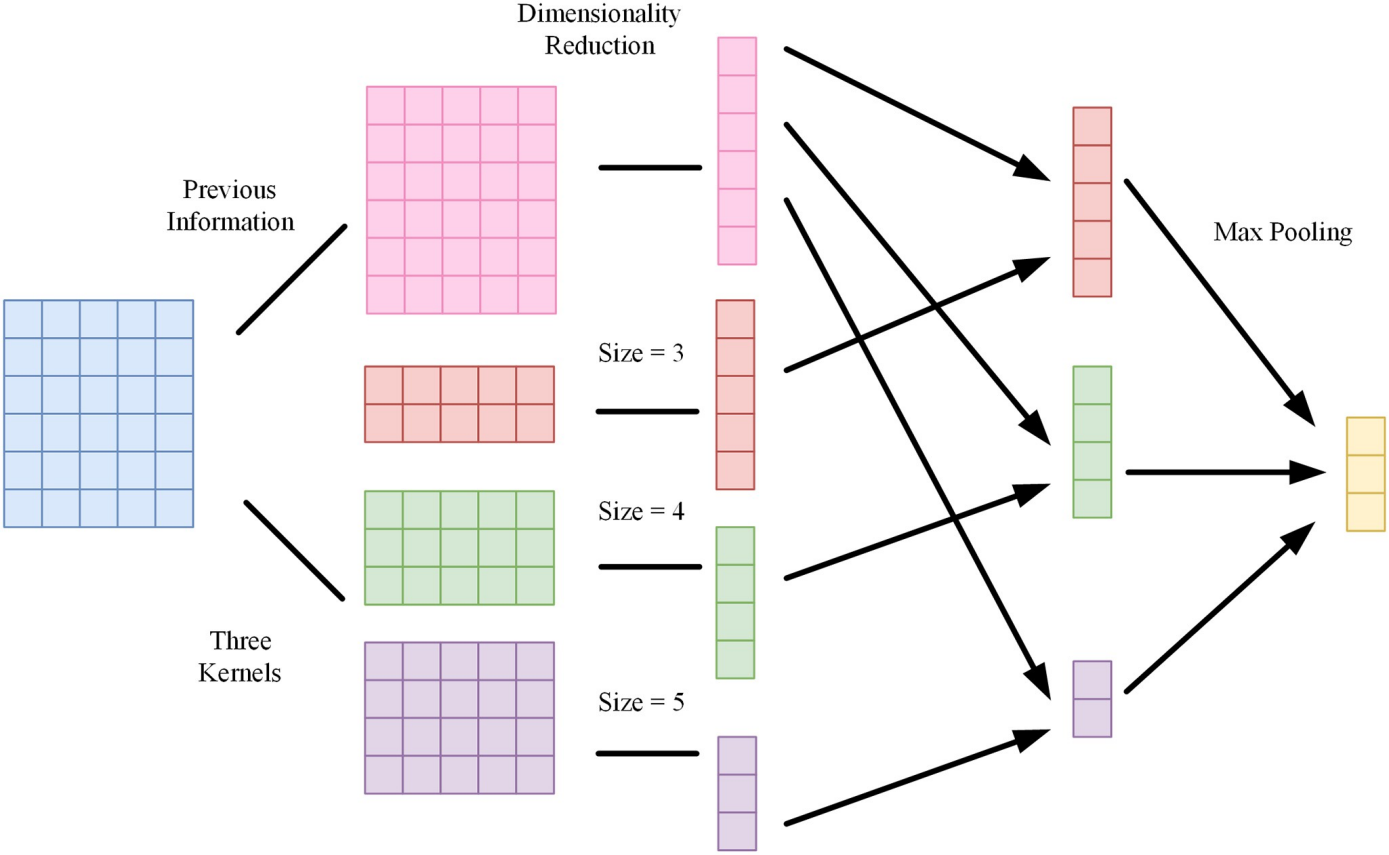
Local semantic module process.

The reordering of statements and functions is a common technique in code plagiarism, and the order of functions can affect feature extraction. If NLP models are used to extract features from binary functions, the results may not be optimal. Convolutional kernels, on the other hand, can extract local contextual features from sliding windows that are not affected by the position of occurrence, which aligns with the requirements of binary code similarity detection. Convolutional Neural Networks (CNNs) have found widespread application across diverse domains within the field of deep learning [30–37]. In text processing, TextCNN uses convolutional kernels of different sizes to extract n-gram features at different positions, obtaining semantic features at various levels. However, its drawback is that it can only capture local relationships in the text, ignoring the impact of long-distance semantics. To address this issue, we introduce local semantic module, which is based on the understanding that semantic comprehension proceeds sequentially from front to back. Therefore, the information before the convolution operation on the instruction embeddings is crucial. In local semantic module, the performance of the model is enhanced by continuously incorporating previous information during the convolution operation; the process is illustrated in Fig 4.

First, embedding vectors are obtained using instruction embedding module, and the previous semantic matrix *R* = {*r*_0_, *r*_1_, ⋯, *r*_*n*_} is generated based on the embedding matrix *S* = {*s*_1_, *s*_2_, ⋯, *s*_*n*_}, as expressed by Eq (6):

$$r_{i}=\frac{\sum_{j=1}^{i}s_{i}}{i}$$ (6)

where *r*_0_ is a zero vector. Subsequently, dimensionality is reduced through a fully connected layer to obtain the previous information vector G = {*g*_0_, *g*_1_, ⋯, *g*_*n*_}. Convolution operations are then performed using convolution kernels *W* of sizes 3, 4, and 5. In each convolution operation, features *c*_*i*_ are obtained, as expressed by Eq (7):

$$c_{i}=f\left(W_{h}*S_{i:i+h-1}+b_{h}\right)$$ (7)

where h*d represents the size of the convolution *W*_*h*_, *d* is the dimension of token embedding, *f* is a nonlinear activation function, *b*_*h*_ is a bias term, and *S*_*i*:*i*+*h*−1_ is the local code matrix from the *i*-th to the (*i* + *h* − 1)-th row of *S*. Finally, the local features are fused with the previous information features to obtain the convolution result *u*_*i*_. Max pooling is applied to *U* = {*u*_1_, *u*_2_, ⋯, *u*_*i*+*h*−1_} using the following formulas:

$$u_{i}=ac_{i}+bg_{i-1}$$ (8)

$$M=\text{max}\;\left\{U\right\}$$ (9)

As bidirectional LSTM takes input in a sequential structure and pooling disrupts the sequential structure of *U*, a fully connected layer is added to concatenate *M*_*i*_ from the pooling layer into a vector *Q*:

$$Q=\left\{M_{1},M_{2},\cdots ,M_{n}\right\}$$ (10)

Subsequently, the new concatenated high-order vector *Q* serves as the input for bidirectional LSTM.

#### 3.3.2 Semantic association module

LSTM is a variant of RNN that addresses issues related to long-range information loss, gradient vanishing, and exploding gradients. It is particularly well-suited for handling sequential information. LSTM analyzes input information using time sequences and incorporates forget, input, and output gates. The calculations for the forget gate *f*_*t*_, memory gate *i*_*t*_, output gate *o*_*t*_, temporary memory state ${\tilde{C}}_{t}$, current memory state *C*_*t*_, and current hidden state *h*_*t*_ are formulated as follows:

$$f_{t}=\sigma \left(W_{f}x_{t}+U_{f}h_{t-1}+b_{f}\right)$$ (11)

$$i_{t}=\sigma \left(W_{i}x_{t}+U_{i}h_{t-1}+b_{i}\right)$$ (12)

$$o_{t}=\sigma \left(W_{o}x_{t}+U_{o}h_{t-1}+b_{o}\right)$$ (13)

$${\tilde{C}}_{t}=\text{tanh}\left(W_{c}x_{t}+U_{c}h_{t-1}+b_{c}\right)$$ (14)

$$C_{t}=i_{t}*{\tilde{C}}_{t}+f_{t}*C_{t-1}$$ (15)

$$h_{t}=o_{t}*\text{tanh}\left(C_{t}\right)$$ (16)

where *W*_*f*_, *U*_*f*_, *W*_*i*_, *U*_*i*_, *W*_*c*_, *U*_*c*_, *W*_*o*_, *U*_*o*_ are the respective weight matrices for the connections, σ denotes the sigmoid function, and *b*_*f*_, *b*_*i*_, *b*_*c*_, *b*_*o*_ are bias vectors for the different stages.

LSTM can only capture previous information, and lacks integration of contextual information in the reverse direction, neglecting information from the posterior context. To achieve more comprehensive feature extraction of instruction vectors and improve result accuracy, bidirectional LSTM is employed to better capture contextual information from both directions, acquiring richer semantic features of binary code. Bidirectional LSTM, which is composed of two LSTM layers in opposite directions, merges the hidden states of the two LSTMs to obtain the output of bidirectional LSTM, enabling access to future contextual information. Bidirectional LSTM is utilized to extract contextual information from local semantic module separately, yielding the forward feature sequence ${\vec{h}}_{t}$ and backward feature sequence ${\overset{\leftarrow }{h}}_{t}$. The calculation formula for the instruction feature vector *h*_*w*_ output by bidirectional LSTM is as follows:

$$h_{w}={\vec{h}}_{t}⊕{\overset{\leftarrow }{h}}_{t}$$ (17)

After bidirectional LSTM, a self-attention layer is added to calculate the weight coefficients for each instruction in the function. This mechanism learns the relationships between instructions within a function, grasps structural information in the code, and enhances the capture of long-range information in the instructions. Self-attention can be formulated as follows:

$$Self-Attention\left(Q,K,V\right)=soft\;\text{max}\left(\frac{QK^{T}}{\sqrt{d}}\right)\cdot V$$ (18)

Finally, the function information undergoes processing through a fully connected layer and activation function, resulting in vector representations for the two binary functions.

### 3.4 Improved cosine similarity

**Fig 5:**
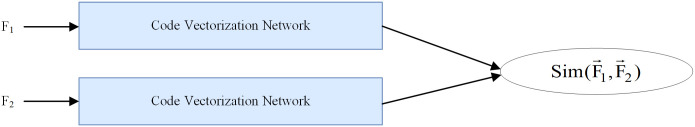
Siamese network.

Siamese is employed to analyze the similarity between two comparable entities, and a crucial component of this architecture is the distance function that represents the similarity. The performance of the model is significantly affected by the choice of the distance function. Methods like Gemini and SAFE measure the similarity between corresponding binary code by computing the cosine similarity between the vectors representing the two functions. Traditional cosine similarity primarily considers the direction of data vectors and does not account for values, making it unable to distinguish cases where the values of various dimensions in two vectors are proportionally scaled. To address this challenge, we improve traditional cosine similarity from the perspective of data values by proposing improved cosine similarity, which utilizes the Hellinger distance [38] and the difference in values taken by different data objects along the same dimension to enhance traditional cosine similarity, As illustrated in Fig 5. The calculation of improved cosine similarity is given by Eq (19):

$$Similarity\left(V,W\right)=\frac{\sum_{i=1}^{m}\sqrt{v_{i}w_{i}}}{\sqrt{\sum_{i=1}^{m}v_{i}}\sqrt{\sum_{i=1}^{m}w_{i}}\sqrt{\sum_{i=1}^{m}\left|v_{i}-\left.w_{i}\right|\right.+1}}$$ (19)

where *V* = (*v*_1_, *v*_2_, ⋯, *v*_*m*_) and *W* = (*w*_1_, *w*_2_, ⋯, *w*_*m*_) are two *m*-dimensional data vectors.

Improved cosine similarity considers the data values in the similarity calculation, addressing the drawback of traditional cosine similarity’s insensitivity to numerical values. This method retains the advantages of traditional cosine similarity in directional discrimination, while also distinguishing data objects with proportionally scaled values in various dimensions. As a result, it achieves more accurate measurements of code similarity.

### 3.5 Loss function

In this paper, pairs of similar and dissimilar code snippets are used as inputs to train the Siamese network, cross-entropy is chosen as the loss function. For each input pair, the probability *p* is utilized to predict the similarity. Loss function is given by

$$Loss=\frac{1}{N}\sum_{i}-\left[y_{i}\cdot \text{log}\;p_{i}+\left(1-y_{i}\right)\cdot \text{log}\left(1-p_{i}\right)\right]$$ (20)

where *y*_*i*_ represents the label of sample *i*, where similarity is denoted as 1 and dissimilarity as −1; and *p*_*i*_ denotes the predicted probability of *i* being similar.

### 3.6 Algorithm steps

Step 1: Collect binary files for preprocessing. Extract the assembly instruction sequences of binary functions using disassembly tools, and input the instructions into the instruction embedding module. Utilize the instruction embedding module to embed the instruction sequences of the two input binary functions into a vector space, converting assembly instructions into real-valued vectors.
Step 2: Input the obtained instruction vectors into the local semantic module. After undergoing convolutional operations with multiple different-sized kernels, extract key features and deeper structural information from the binary functions.
Step 3: Perform max pooling on each feature map obtained from the local semantic module in the pooling layer, resulting in unified feature values. Obtain hidden vectors of fixed length.
Step 4: Utilize the semantic association module to capture long-distance global feature information of binary code. Employ self-attention mechanism to calculate the weight coefficients of each instruction in the function, assigning different weights to instruction vectors, thus learning inter-function dependencies.
Step 5: The function information undergoes processing through a fully connected layer and activation function, resulting in vector representations for the two binary functions.
Step 6: Utilize an improved cosine similarity method to calculate the distance between the embedding vectors of two binary functions, outputting the similarity between the two binary functions.

## 4. Experimental setup

### 4.1 Datasets

To compare our model with existing methods, we created three binary code datasets, having different architectures, different compilers, and different optimization levels. We chose x64, x86, and ARM as the different architectures, Clang and GCC as the different compilers, and O0, O1, O2, and O3 as the different optimization levels. The same source code was compiled into different binary files in various compilation environments. After compilation, we used the ANGR binary analysis Python framework to disassemble the binary code, extract basic blocks, and label binary functions along with their names. In total, the cross-architecture dataset consisted of 128,396 functions, the cross-compiler dataset consisted of 102,682 functions, and the cross-optimization level dataset consisted of 203,738 functions.

### 4.2 Dataset processing

After the datasets were obtained, preprocessing was essential for the binary code similarity detection task. The objective of the task was to determine whether two functions are similar, requiring the generation of pairs of functions. Each function pair consisted of two binary functions and a label indicating whether they are similar. In this study, there were two types of pairs: similar function pairs and dissimilar function pairs. Similar function pairs were associated with a label of +1, while dissimilar function pairs were associated with a label of −1. The dataset was divided into a training set, validation set, and test set in an 8:1:1.

### 4.3 Hyperparameter selection

The experiments were conducted on the Windows 10 operating system, utilizing an NVIDIA RTX 3090 GPU and the PyCharm development platform.

Regarding the hyperparameters, in the pretraining phase for the assembly instruction representation method, as the average length of assembly instructions is smaller than the average length of sentences in natural language, the number of attention heads for instruction embedding module was reduced from 12 (as in BERT) to 8. The token embedding dimension was 256, and the other parameters remained unchanged. For local semantic module, window sizes of 3, 4, and 5 with a stride of 1 were used. The hyperparameter table for training the model is as follows in Table 1:

**Table 1:** Hyperparameter of model.

Hyperparameters	Values
Embedding dimension	256
Number of head	8
Hidden size	256
Dropout	0.1
Learning rate	0.0005
Optimizer	Adam
Epoch	60
Batch size	32

### 4.4 Evaluation metrics

The following evaluation metrics are commonly used to assess the model’s performance, where *TP*, *FP*, *TN*, and *FN* represent the counts of true positive, false positive, true negative, and false negative, respectively.


$$Accuracy=\frac{TP+TN}{TP+TN+FN+FP}$$ (21)

$$Percision=\frac{TP}{TP+FP}$$ (22)

$$F1=2*\frac{Precision*Recall}{Precision+Recall}$$ (23)

## 5. Evaluation

We selected Ins2vec [22], SAFE [1], and TCCCD [23] as baselines. The evaluations of different methods were conducted in three experiments involving different architectures, compilers, and optimization levels.

Ins2vec [22] is a straightforward neural network architecture based on bidirectional gate recurrent units. It employs an assembly language model utilizing length-ratio shuffle and skip-gram with negative sampling for instruction embedding, focusing solely on the semantic information of basic blocks. These embeddings are subsequently fed into a Siamese architecture to learn basic block embeddings.

SAFE [1] acts directly on disassembled binary functions without the need for manual feature extraction, enabling rapid generation of embeddings for hundreds of binary files via the Word2Vec model. This approach utilizes bidirectional gate recurrent units to capture the sequential interactions of instructions by considering both the instructions themselves and the context of the function. Furthermore, the Self-Attentive Network disregards noise and redundant information in the input, focusing on features crucial for the detection outcome.

TCCCD [23] initially represents code as abstract syntax trees (ASTs) and segments the ASTs into syntactic subtrees, thereby encapsulating the hierarchical structure and information of the code. Subsequently, in terms of neural network architecture, TCCCD employs the Encoder part of the Transformer to extract the global information of the code, and then utilizes convolutional neural networks to capture the local information of the code. Finally, it integrates features extracted from both networks to learn code vector representations imbued with lexical, syntactic, and structural information.

### 5.1 Different architectures

In this experiment, we utilized the same compiler, GCC-8.2.0, and the identical optimization level, O0. x86, x64, and x86 function pools were separately used as target function pools, with x64, ARM, and ARM function pools as function pools for target function search. The experimental results for three different architectures—x86 and x64, x64 and ARM, x86 and ARM—are presented in Tables 2–4.

**Table 2:** Accuracy of different architectures.

Method	x86-x64	x64-ARM	x86-ARM
Ins2vec	0.817	0.791	0.795
SAFE	0.884	0.873	0.871
TCCCD	0.892	0.891	0.885
BinBcla	0.924	0.917	0.922

**Table 3:** Precision of different architectures.

Method	x86-x64	x64-ARM	x86-ARM
Ins2vec	0.764	0.772	0.769
SAFE	0.826	0.823	0.817
TCCCD	0.853	0.861	0.831
BinBcla	0.915	0.893	0.895

**Table 4:** F1 score of different architectures.

Method	x86-x64	x64-ARM	x86-ARM
Ins2vec	0.782	0.779	0.774
SAFE	0.804	0.805	0.807
TCCCD	0.851	0.853	0.836
BinBcla	0.912	0.885	0.881

In the three sets of experiments involving x86 and x64, x64 and ARM, x86 and ARM, BinBcla’s accuracy was 0.924, 0.917, and 0.922, the precision was 0.915, 0.893, and 0.895, and the F1 score was 0.912, 0.885, and 0.881, respectively. In the experiments, the average accuracy of Ins2vec, SAFE, and TCCCD were 0.801, 0.876, and 0.889, respectively. The average precision values were 0.768, 0.822, and 0.848, respectively. The average F1 score values were 0.778, 0.805, and 0.847, respectively. Our model achieved an average accuracy of 0.921, an average precision of 0.901 and an average F1 score of 0.893. Overall, BinBcla showed an improvement of 14.98%, 5.14%, and 3.60% in accuracy, 17.32%, 9.61%, and 6.25% in precision, and 14.78%, 10.93%, and 5.43% in F1 score compared with Ins2vec, SAFE, and TCCCD, respectively. The results indicate that in cross-architecture experiments, the proposed model demonstrates better resistance to cross-architecture differences and exhibits stronger robustness.

### 5.2 Different compilers

In this experiment, the same architecture (x64) and identical optimization level (O0) were employed. Clang-4.0, GCC-5.5.0, and Clang-7.0 function pools were, respectively, used as the target function pools; and Clang-7.0, GCC-8.2.0, and GCC-8.2.0 function pools served as the target function search pools. In the domain of binary code similarity, varying versions of compilers are considered as different compilers. The results of the three experimental sets are presented in Tables 5–7.

**Table 5:** Accuracy of different compilers.

Method	Clang-4.0 & Clang-7.0	GCC-5.5.0 & GCC-8.2.0	Clang-7.0 & GCC-8.2.0
Ins2vec	0.829	0.836	0.823
SAFE	0.903	0.907	0.885
TCCCD	0.924	0.916	0.907
BinBcla	0.932	0.947	0.943

**Table 6:** Precision of different compilers.

Method	Clang-4.0 & Clang-7.0	GCC-5.5.0 & GCC-8.2.0	Clang-7.0 & GCC-8.2.0
Ins2vec	0.808	0.826	0.814
SAFE	0.861	0.859	0.843
TCCCD	0.895	0.862	0.867
BinBcla	0.903	0.931	0.917

**Table 7:** F1 score of different compilers.

Method	Clang-4.0 &Clang-7.0	Gcc-5.5.0 &Gcc-8.2.0	Clang-7.0 &Gcc-8.2.0
Ins2vec	0.785	0.818	0.797
SAFE	0.870	0.874	0.851
TCCCD	0.884	0.872	0.875
BinBcla	0.901	0.925	0.908

It can be observed that in the experiments involving Clang-4.0 and Clang-7.0, GCC-5.5.0 and GCC-8.2.0, Clang-7.0 and GCC-8.2.0, BinBcla’s accuracy was 0.932, 0.947, and 0.943, respectively, with precision values of 0.903, 0.931, and 0.917 and F1 score values of 0.901, 0.925, and 0.908. In the experiments, the average accuracy of Ins2vec, SAFE, and TCCCD were 0.829, 0.898, and 0.916, respectively. The average precision values were 0.816, 0.854, and 0.875, respectively. The average F1 score values were 0800, 0.865, and 0.877, respectively. Our model achieved an average accuracy of 0.941, an average precision of 0.917 and an average F1 score of 0.911. Overall, compared to Ins2vec, SAFE, and TCCCD, BinBcla exhibited improvements in accuracy of 13.51%, 4.79%, and 2.73%, in precision of 12.38%, 7.38%, and 4.80%, and in F1 score of 13.88%, 5.32%, 3.88%, respectively, in experiments involving different compilers. These results indicate that in cross-compiler experiments, the proposed model demonstrates greater resistance to cross-compiler variations, achieving higher accuracy, precision and F1 score.

### 5.3 Different optimization levels

In this experiment, the dataset with cross-optimization levels was utilized, using the same x64 architecture and the Clang-7.0 compiler. The target function pools were formed using functions compiled with O0, O0, O0, O1, O1, and O2 optimization levels, while the search function pool consisted of functions compiled with O1, O2, O3, O2, O3, and O3 optimization levels. The results of six different optimization levels are presented in Tables 8–10.

**Table 8:** Accuracy of different optimization levels.

Method	O0-O1	O0-O2	O0-O3	O1-O2	O1-O3	O2-O3
Ins2vec	0.811	0.814	0.803	0.835	0.819	0.827
SAFE	0.896	0.883	0.854	0.887	0.895	0.908
TCCCD	0.913	0.912	0.877	0.917	0.903	0.894
BinBcla	0.941	0.933	0.916	0.934	0.928	0.936

**Table 9:** Precision of different optimization levels.

Method	O0-O1	O0-O2	O0-O3	O1-O2	O1-O3	O2-O3
Ins2vec	0.729	0.768	0.683	0.726	0.702	0.741
SAFE	0.823	0.827	0.769	0.834	0.796	0.793
TCCCD	0.871	0.835	0.832	0.843	0.859	0.854
BinBcla	0.923	0.904	0.901	0.929	0.907	0.913

**Table 10:** F1 score of different optimization levels.

Method	O0-O1	O0-O2	O0-O3	O1-O2	O1-O3	O2-O3
Ins2vec	0.716	0.749	0.692	0.714	0.701	0.736
SAFE	0.828	0.805	0.761	0.826	0.784	0.791
TCCCD	0.883	0.851	0.845	0.869	0.848	0.879
BinBcla	0.920	0.895	0.893	0.917	0.892	0.915

In the experiments, BinBcla’s accuracy was 0.941, 0.933, 0.916, 0.934, 0.928, and 0.936, respectively. The precision values were 0.923, 0.904, 0.901, 0.929, 0.907, and 0.913, respectively. The F1 score values were 0.920, 0.895, 0.893, 0.917, 0.892, and 0.915, respectively. In the experiments, the average accuracy of Ins2vec, SAFE, and TCCCD were 0.818, 0.887, and 0.903, respectively. The average precision values were 0.725, 0.807, and 0.849, respectively. The average F1 score values were 0.718, 0.799, and 0.863, respectively. Our model achieved an average accuracy of 0.931, an average precision of 0.913, and an average F1 score of 0.905. Overall, BinBcla demonstrated improvements in accuracy of 13.81%, 4.96%, 3.10% relative to Ins2vec, SAFE, and TCCCD, improvements in precision of 25.93%, 13.14%, 7.54%, and improvements in F1 score of 26.04%, 13.27%, 4.87%, respectively. In all six sets of the experiments, our model achieved the highest accuracy, precision and F1 score among all the models, indicating that in cross-optimization level experiments, our model is more robust to cross-optimization level differences and exhibits superior performance.

### 5.4 Ablation studies

In the ablation experiments, we investigated several factors influencing the detection performance of the model, including the embedding model, local semantic module, and the distance function.

In the ablation experiments, the x64 architecture was retained, and the compilation optimization level was O0. We used Clang-7.0’s function pool as the target function pool and GCC-8.2.0’s function pool as the search function pool for the target functions. In Model 1, instruction embedding module was replaced with Word2Vec and the other components were unchanged. In Model 2, local semantic module was replaced with CNN and the other components were unchanged. In Model 3, improved cosine similarity was replaced with traditional cosine similarity and the other components were unchanged. In Model 4, local semantic module was removed and the other components were unchanged. Model 5 represents our proposed model. The results are shown in Table 11.

**Table 11:** Ablation experiment.

Method	Accuracy	Precision	F1 score
Model 1	0.786	0.728	0.742
Model 2	0.925	0.906	0.890
Model 3	0.912	0.873	0.875
Model 4	0.916	0.894	0.891
Model 5	0.943	0.917	0.908

1. In the experiments evaluating the embedding models, keeping the other components unchanged, we only varied the embedding model. The accuracy using the Word2Vec model was 0.786, precision was 0.728, and F1 score was 0.742. In contrast, our model, utilizing instruction embedding module, achieved an accuracy of 0.943, precision of 0.917, and F1 score was 0.908. This suggests that instruction embedding module can dynamically adjust token vectors based on different contextual information, addressing the issue of polysemy that Word2Vec struggles with. This adaptation capability contributes to improved accuracy, precision and F1 score in the model.
2. In the experiments evaluating local semantic module, while keeping the other components unchanged, we replaced local semantic module with standard CNN. The model using CNN achieved an accuracy of 0.925, precision of 0.906 and F1 score was 0.890. Our model, incorporating local semantic module, achieved an accuracy of 0.943, precision of 0.917 and F1 score was 0.908. This validates that local semantic module, by not only focusing on local information features, but also incorporating previous information, contributes to enhancing the model’s performance. When local semantic module was removed and the other components were unchanged, the model achieves an accuracy of 0.916, a precision of 0.894 and F1 score was 0.891. This validates that multi-level semantic feature extraction can obtain more accurate code semantic information.
3. In the experiments evaluating the distance function, while keeping the other components unchanged, we replaced improved cosine similarity with traditional cosine similarity. The model using traditional cosine similarity achieved an accuracy of 0.912, precision of 0.873 and F1 score was 0.875. Our model, employing improved cosine similarity, achieved an accuracy of 0.943, precision of 0.917 and F1 score was 0.908. This indicates that improved cosine similarity better captures the relationships between all elements in the distance vector, fundamentally enhancing the model’s robustness to new sample functions, making complex inferences feasible, and improving overall performance.

### 5.5 Hyperparameter experiment

This section discusses the impact of hyperparameters on BinBcla’s performance, focusing on the number of epochs and the dimensions of function embeddings. In the hyperparameter experiments, we maintained a consistent architecture (x64) and optimization level (O0). The target function pool was derived from functions compiled with Clang-7.0, and the function pool for searching was based on functions compiled with GCC-8.2.0.

**Fig 6:**
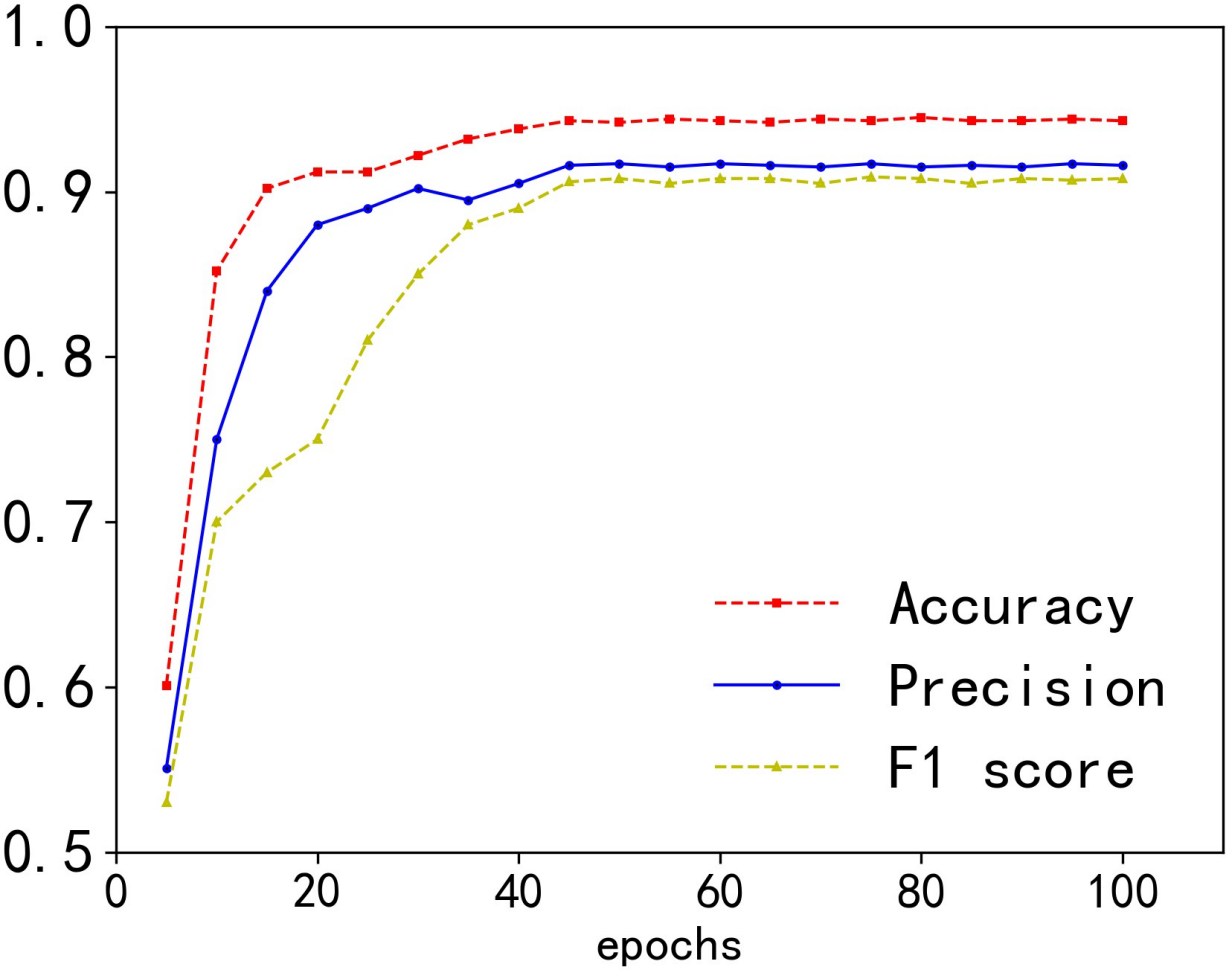
Impact of epochs.

**Fig 7:**
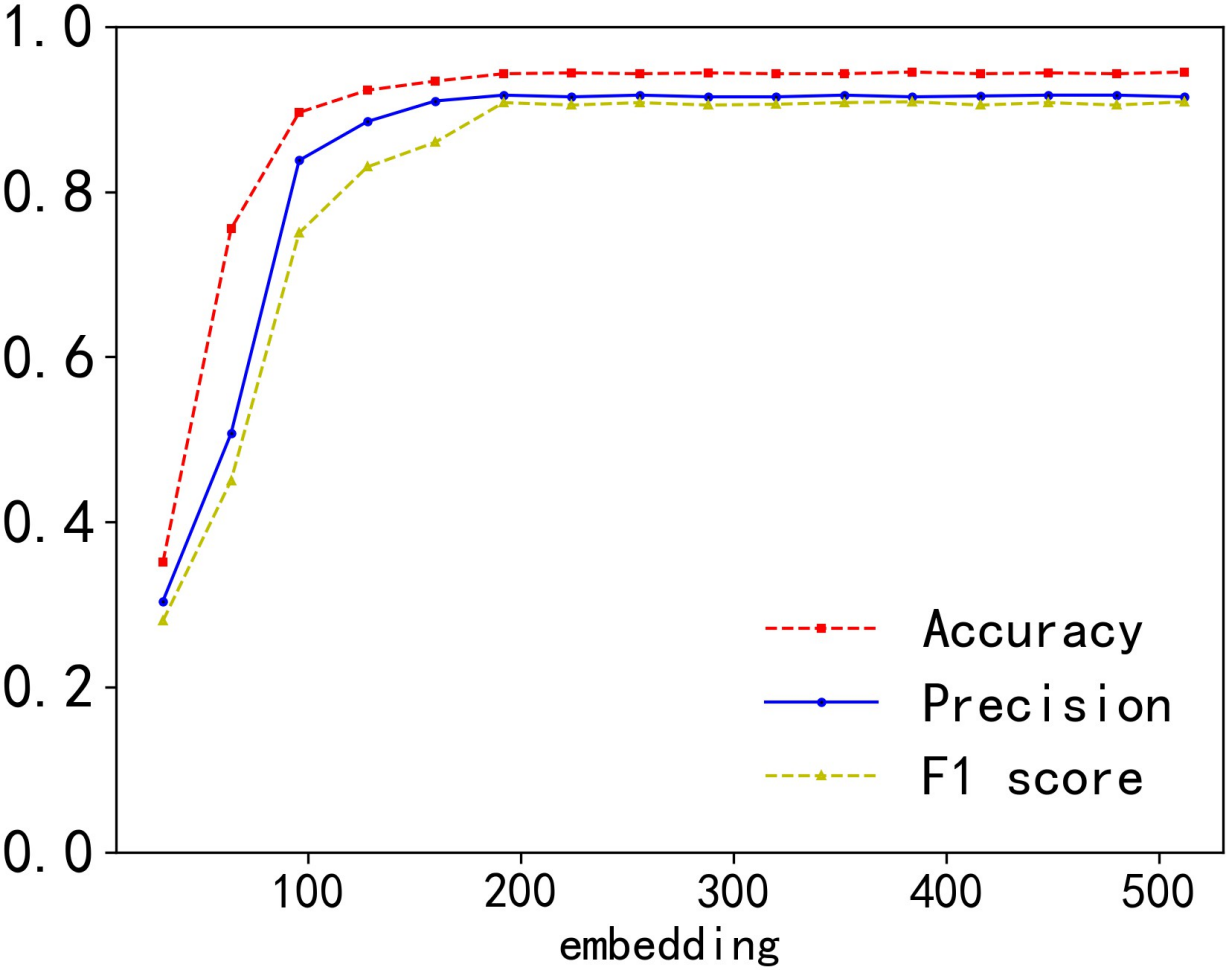
Impact of embedding.

1. The number of epochs is crucial in training the model. If the number is too low, the model may not have sufficient training time. However, if the number is too high, the model might face overfitting. To determine the optimal number of epochs, we trained the model with 100 epochs and then evaluated its performance. As depicted in Fig 6, our model’s accuracy, precision and F1 score became relatively stable around the 60th epoch. Moreover, after stability was achieved, there was no degradation in performance metrics with a continuous increase in epochs, indicating that our model exhibits better robustness.
2. The dimension of function embeddings is another critical hyperparameter that we examined. We conducted tests using different embedding dimensions. Intuitively, an embedding vector with a lower dimension contain relatively less semantic information, potentially leading to decreased model performance. As illustrated in Fig 7, when the embedding dimension was set to 32, the accuracy, precision and F1 score of the model were relatively low. With an increase in embedding dimension, the accuracy, precision and F1 score continued to rise. However, when the embedding dimension surpassed 256, there was almost no further increase in accuracy, precision and F1 score. Additionally, an excessively large embedding dimension can result in higher memory usage and longer training time. Therefore, considering the balance between effectiveness and resource constraints, we selected 256 as the optimal embedding dimension.

### 5.6 Limitations

1. Function Inlining: The purpose of function inlining is to reduce the runtime overhead incurred when entering and exiting functions. However, it can result in significant changes to the assembly program, posing challenges for labeling based on function names, as binary functions with similar semantics may be classified as dissimilar. In the future, we will further investigate this issue.
2. Code Obfuscation: Our model primarily focuses on analyzing the similarity of binary code without applying code obfuscation techniques. This limitation means that our model cannot directly be applied to binary code that has undergone code obfuscation. However, in the future, we plan to conduct research in this area to expand the capabilities of our model.

## 6. Conclusion

In this paper, we proposed a novel binary code similarity detection model, called BinBcla. Firstly, considering the differences between binary code and natural language. To further enhance code representation, we develop an instruction embedding module with a newly designed training task, based on an enhanced BERT architecture, to provide dynamic semantic embeddings for binary code instructions. It dynamically adjusts token embedding based on varying contextual information, addressing the challenge of handling polysemy that Word2Vec struggles with. Secondly, to address the issue of insufficient semantic information extraction by a single neural network model, we propose a multi-feature fusion technique that performs feature extraction at different levels on binary code, greatly enriching the semantic feature information of binary code. Finally, considering traditional cosine similarity being unable to distinguish differences between data objects with proportionally changing values in various dimensions, we propose an improved cosine similarity method that learn the distance vectors, understanding relationships among all elements of the distance vectors. Through experimental evaluation, our proposed method demonstrated superior performance across different architectures, compilers, and optimization levels compared with previous models. This result demonstrates the effectiveness and advancement of the proposed method.

In the future, we plan to integrate graph structure-aware embedding with semantic information to further enhance the model’s performance. Additionally, we will conduct experiments related to code obfuscation detection.
